# Conceptual and relational advances of the PARIHS and i-PARIHS frameworks over the last decade: a critical interpretive synthesis

**DOI:** 10.1186/s13012-022-01254-z

**Published:** 2022-12-07

**Authors:** Yinfei Duan, Alba Iaconi, Jing Wang, Janelle Santos Perez, Yuting Song, Stephanie A. Chamberlain, Shovana Shrestha, Katharina Choroschun, Matthias Hoben, Anna Beeber, Ruth A. Anderson, Greta G. Cummings, Holly J. Lanham, Peter G. Norton, Carole A. Estabrooks, Whitney Berta

**Affiliations:** 1 Faculty of Nursing, University of Alberta, Edmonton, Alberta Canada; 2 School of Nursing, University of North Carolina at Chapel Hill, Chapel Hill, NC USA; 3 School of Nursing, Qingdao University, Qingdao, Shandong China; 4 School of Public Health, Bielefeld University, Bielefeld, Germany; 5 School of Health Policy and Management, Faculty of Health, York University, Toronto, Ontario Canada; 6 School of Nursing, Johns Hopkins University, Baltimore, MD USA; 7 Joe R. & Teresa Lozano Long School of Medicine, University of Texas Health, San Antonio, TX USA; 8 Department of Family Medicine, University of Calgary, Calgary, Alberta Canada; 9 Institute of Health Policy, Management and Evaluation, University of Toronto, Toronto, Ontario Canada

## Abstract

**Background:**

The number of research publications reporting the use of the Promoting Action on Research Implementation in Health Services (PARIHS) framework and the integrated PARIHS (i-PARIHS) framework has grown steadily. We asked how the last decade of implementation research, predicated on the (i-)PARIHS framework (referring to the PARIHS or i-PARIHS framework), has contributed to our understanding of the conceptualizations of, relationships between, and dynamics among the core framework elements/sub-elements. Building on the Helfrich et al*.* (2010) review of research on the PARIHS framework, we undertook a critical interpretive synthesis to: (1) identify conceptual and relational advances in the (i-)PARIHS framework and (2) identify conceptual and relational aspects of the (i-)PARIHS framework that warrant further work.

**Methods:**

We performed a systematic search in PubMed/PubMed Central, Ovid MEDLINE, CINAHL, JSTOR, SCOPUS, Web of Science, and PsycInfo. Articles were eligible for synthesis if they (a) were peer-reviewed articles, written in English, and published between January 2009 and December 2021, (b) applied the (i-)PARIHS framework explicitly to guide implementation research, and (c) made conceptual (expanding the conceptualization of core elements) and/or relational contributions (elaborating relationships among elements/sub-elements, or theorizing the relationships using empirical data). We used a critical interpretive synthesis approach to synthesize conceptual-relational advances of the (i-)PARIHS framework.

**Results:**

Thirty-seven articles were eligible for synthesis. Twenty-four offered conceptual contributions, and 18 offered relational contributions (5 articles contributed in both ways). We found conceptual expansion of all core (i-)PARIHS elements, with most emphasis on *context* (particularly *outer context* and *leadership*)*, facilitation,* and *implementation success.* Articles also gave insights into the complex relationships and relational dynamism among these elements, characterized as contingent, interactive, multilevel, and temporal effects.

**Conclusions:**

We observed developmental advances of the (i-)PARIHS framework and proposed several directions to further advance the framework. Conceptualization of (i-)PARIHS elements (particularly *evidence/innovation* and *recipients*) need to be further developed by specifying conceptual and operational definitions of underlying sub-elements. Relationships among (i-)PARIHS elements/sub-elements need to be further elaborated through empirical studies that consider situational contingencies and causal complexities. This will require examining necessity and sufficiency of (i-)PARIHS elements/sub-elements in relation to implementation outcomes, interactions among elements, and mechanism-based explanations.

**Supplementary Information:**

The online version contains supplementary material available at 10.1186/s13012-022-01254-z.

Contributions to the literature • By summarizing the last decade of literature, this review reveals an increasing number of studies that explicitly applied the Promoting Action on Research Implementation in Health Services (PARIHS) or i-PARIHS framework to guide implementation research.

• Although most of the studies only confirmed established aspects of the framework, we identify some studies that offer conceptual and relational advances by expanding the conceptualization of core elements of the framework and empirically testing complex relationships among elements.

• Findings also suggest theoretical and methodological considerations for researchers to advance the framework in terms of the conceptualizations of and relationships among elements of the framework.

## Introduction

The Promoting Action on Research Implementation in Health Services (PARIHS) framework was first introduced in 1998. Since then, it has been used extensively by implementation science researchers and practitioners to predict or explain successful implementation of evidence-based healthcare initiatives [1–3]. Authors of the original PARIHS framework contended that successful implementation of evidence-based practices was a function of *evidence* [4], *context* [5], and *facilitation* [6]. Accordingly, successful implementation is more likely when (a) robust evidence matches practitioner, patient, and local experiences; (b) context is receptive to change with sympathetic cultures, effective leadership, and appropriate evaluative systems; and (c) skilled facilitators provide appropriate facilitation of change [7]. While it is applied extensively, the PARIHS framework has been criticized for challenges related to applying it [8–10]. In their review of the PARIHS literature, Helfrich et al. [9] noted that (a) the PARIHS elements/sub-elements lack conceptual clarity, (b) definitions of the key outcome *successful implementation* vary, and (c) those who developed and applied the PARIHS framework rarely examine relationships among elements/sub-elements. Another critique was that PARIHS was largely used as an organizing or heuristic device, and often retrospectively rather than prospectively [8–10]. This limits the framework’s potential to guide diagnostic analysis of evidence and context, and to inform design of facilitation/implementation strategies.

Some of the framework’s original authors published the integrated PARIHS framework (i-PARIHS) in 2016 to address concerns over conceptual clarity and describe the framework’s theoretical antecedents [11]. The main changes to the framework were (a) including intended *recipients* of implementation as a new core concept; (b) enhancing the description of *evidence* (now identified as *innovation*, given that new knowledge is usually introduced to generate change or improvement); (c) elaborating the definition of *context* to differentiate inner and outer context; and (d) refining the concept of *facilitation* by dividing facilitator roles into beginner, experienced, or expert facilitators and acknowledging internal or external facilitators [11, 12]. The i-PARIHS framework also sets forth interconnections of the core elements by “positioning *facilitation* as the active ingredient of implementation, assessing and aligning the *innovation* to be implemented with the intended *recipients* in their local, organizational, and wider system *context*.” [11]. It is not clear if these adaptations have supported more effective application of the framework in implementation research and practices.

The number of research publications reporting the use of the (i-)PARIHS framework (referring to the PARIHS or i-PARIHS framework), has grown steadily since the Helfrich et al. [9] review in 2010, according to citation analysis [3]. Building on the Helfrich et al. [9] review, we undertook a critical interpretive synthesis. We asked how the last decade of implementation research, predicated on the (i-)PARIHS framework, has contributed to our understanding of the conceptualizations of, relationships between, and dynamics among the main framework elements/sub-elements. Specifically, the aims of this synthesis were to (1) identify conceptual and relational advances in the (i-)PARIHS framework and (2) identify conceptual and relational aspects of the (i-)PARIHS framework that warrant further work.

## Methods

We used a critical interpretive synthesis approach because our objectives were descriptive and interpretive rather than aggregative as intended by conventional systematic reviews [13]. This approach was also that applied in Helfrich et al. [9]’s review, from which ours built. The focus of the current review is to answer questions: *How were (i-)PARIHS elements/sub-elements conceptualized*? And, *Were conceptual-relational understandings of the framework advanced*? A critical interpretive synthesis approach allowed us to critically reflect on evidence related to conceptualizing and theorizing the (i-)PARIHS framework drawn from a diverse body of literature (including both qualitative and quantitative research) and to produce a descriptive and interpretive account of the final findings.

This review did not involve an appraisal of the methodological quality of included studies. While conventional systematic reviews evaluate against methodological standards designed for specific study designs, a critical interpretive synthesis locates evidence throughout a research article from the study introduction to discussions of research findings and is conceptual in synthesis process and output [13]. A “hierarchy of evidence” approach to evaluating strength of evidence and methodological quality appraisal tools to examine a range of study validity (e.g., internal validity, external validity, statistical validity), as per that employed in conventional systematic reviews, is not applicable for the current review. Our decisions about including articles for the final synthesis prioritized conceptual relevance. The decision-making processes involved recurrent team discussions and reflection, and consensus-seeking processes (as described in the subsequent Methods subsections).

### Search for relevant literature

We performed a systematic search in databases of multiple disciplines, including PubMed/PubMed Central, Ovid MEDLINE, CINAHL, JSTOR, SCOPUS, Web of Science, and PsycInfo. We used a key term search strategy, with key terms including ‘PARIHS,’ ‘promoting action on research implementation in health services,’ and ‘i-PARIHS.’ We restricted the search to English-language publications from January 1, 2009 to December 31, 2021. This work built on Helfrich and colleagues’ 2010 review of literature published before March 2009, so we chose January 2009 as the start date of publication for our search. A health sciences librarian who specialized in knowledge translation and implementation sciences assisted in developing search strategies for each database. Additional file 1: Supplementary file 1 shows search strategies used in each database.

### Article selection

We used predefined inclusion criteria: (a) peer-reviewed articles with full text available, written in English, and published between January 1, 2009 and December 31, 2021; (b) articles making explicit reference to the (i-)PARIHS framework; (c) empirical articles using the (i-)PARIHS framework to inform the development of implementation interventions, measurement or data collection, data analysis, or interpretation or evaluation of study findings; or (d) conceptual articles contributing to conceptual advances of the framework. Articles were excluded if they: (a) did not mention the (i-)PARIHS framework in the text or (b) mentioned or cited the (i-)PARIHS framework but the use of the framework was cursory or peripheral to the focus of the article.

One reviewer (YD) first screened titles and abstracts to remove articles that were not peer-reviewed (e.g., dissertations, theses, or book chapters) and articles with no full text available. Two reviewers (YD and AI) performed dual, independent full-text screening. For each article, they documented how the (i-)PARIHS framework were used and assigned the article to *include*, *exclude*, or *not sure* categories based on our predefined inclusion and exclusion criteria. Screening results from the two reviewers were compared, and articles with conflicting decisions or assigned *not sure* by both reviewers were screened by a third reviewer (JSP or JW). Articles marked for inclusion by at least two reviewers were included for the next step of data extraction.

### Data extraction

We extracted both general information about each article and information specific to application of the (i-)PARIHS framework (see Table 1). Iteratively, we developed a standardized data extraction tool using Microsoft Excel, with detailed instructions on extracting each type of information. Eligible articles were distributed among six reviewers (YD, AI, JSP, JW, YS, WB) for independent data extraction. Before independent coding, all reviewers participated in extensive discussions on the extraction form and fields to ensure a consistent understanding of the data extraction instructions. As a group, reviewers piloted the data extraction form on 20 randomly selected articles. Here, each reviewer independently extracted data from the same set of articles, then the reviewers met as a group to discuss the pilot data extraction. These discussions led to improvements in the clarity of data extraction instructions.

**Table 1:** Data extraction template

**Information extracted from each included article**	
**(1) General information about each article** • Article type • Purposes of the article • Country(ies) • Research setting(s) • Research design (if the article type was a protocol or a report of empirical study)	
**(2) Information specific to the application of PARIHS or i-PARIHS** • How PARIHS or i-PARIHS was used, selecting from ○ Guiding the development of implementation strategies to deliver a clinical intervention, a quality improvement (QI) innovation, or an evidence-based practice project ○ Informing measurement or data collection ○ Informing data analysis ○ Informing the interpretation or evaluation of study findings ○ In other ways • If PARIHS or i-PARIHS was used alone or in combination with other frameworks • Conceptualizations of PARIHS or i-PARIHS core elements and sub-elements (documenting the conceptual and operational definitions, measures of each core element) • Relationships among PARIHS or i-PARIHS elements and sub-elements tested • Comments or discussions around PARIHS or i-PARIHS in the article	

### Analysis

Analysis consisted of coding and interpretive synthesis steps. Each reviewer (YD, AI, JP, JW, YS, WB) coded a sub-set of articles based on the data extraction results, using predefined codes drawing on the (i-)PARIHS framework. Each article was reviewed and independently coded by two reviewers. The codes specified whether the article made conceptual and/or relational contributions to the (i-)PARIHS framework and which elements/sub-elements were involved. Reviewers also gave narrative accounts of the contributions. Articles were considered to make conceptual and/or relational contributions if they: (i) expanded the conceptualization of core (i-)PARIHS elements, (ii) elaborated relationships among (i-)PARIHS elements/sub-elements, or (iii) theorized the relationships using empirical data. Articles were excluded if they only confirmed established aspects of the (i-)PARIHS framework. We excluded articles by the PARIHS developers that focused on revision of the original PARIHS framework. Two reviewers (YD and AI) reviewed the coding results across the reviewer pairs and documented their agreement or disagreement. Discrepancies were discussed in meetings of all reviewers until consensus was reached.

For our final interpretive synthesis, we only included articles that met our criteria for conceptual and/or relational advances. Two reviewers (YD and WB) independently conducted in-depth analysis of conceptual or relational contributions of articles based on the narrative accounts of contributions provided by reviewers in the coding process. YD and WB documented the results thematically according to types of contributions (conceptual, relational) and element/sub-elements of the (i-)PARIHS framework involved. The results were discussed with all other reviewers at recurrent team meetings. The final output, then, incorporated the outcomes of the two independent analyses and inputs/comments from other reviewers.

## Results

**Fig. 1:**
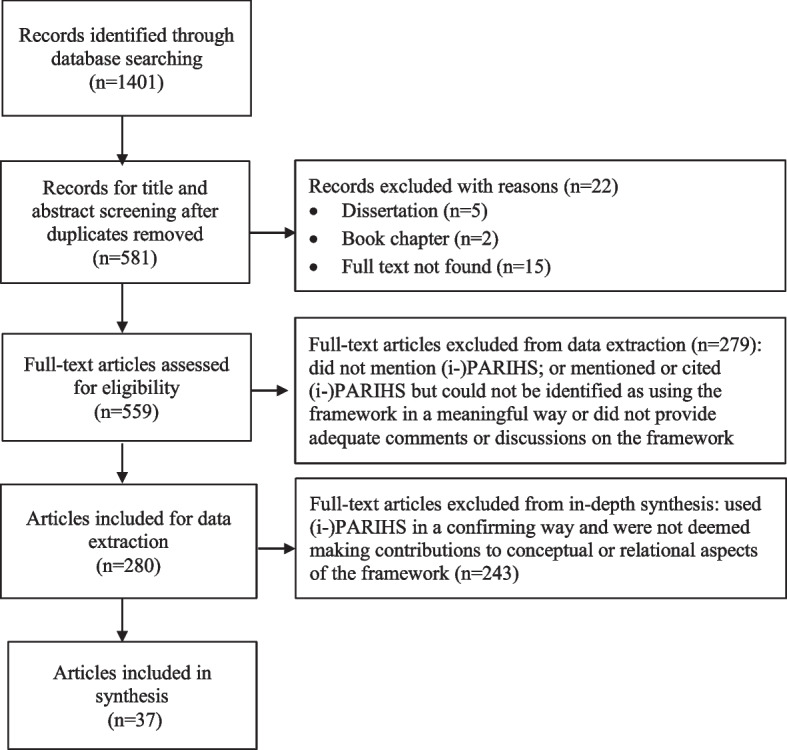
Flow diagram of article selection

Figure 1 shows our article selection process. We identified 280 articles that met the eligibility criteria for data extraction. Of those, 37 articles met the criteria for conceptual and/or relational contributions to the (i-)PARIHS framework. We included these 37 articles in the final interpretive synthesis (Table 2). Of these, 29 were empirical studies (23 qualitative studies, 3 quantitative studies, 3 mixed-methods studies), 4 were literature reviews, and 4 were theory/methodological articles. Except for one study conducted in the construction industry, all empirical studies featuring the (i-)PARIHS were conducted in healthcare settings including acute care/hospitals, long-term care, primary care/community care, and US Veterans Health Administration. Twenty-four offered conceptual contributions, and 18 offered relationships among core elements and/or sub-elements (5 articles contributed in both ways).

**Table 2:** Overview of articles included in the interpretive synthesis (*n* = 37)

Type of article	First author	Year	Country	Setting	Purpose of the study/article	Approach	PARIHS or i-PARIHS used	Application of (i-)PARIHS	Contributions
Empirical study	Carlan [21]	2012	Canada	Other (construction industry)	Determine the most effective ways to disseminate ideas to reduce musculoskeletal disorders in the construction sector	Qualitative	PARIHS	Interpretation	***Conceptual***: context
Empirical study	Bergstrom [22]	2012	Uganda (Africa)	Acute care/hospitals	Examine the relevance of contextual elements perceived to influence knowledge translation	Qualitative	PARIHS	Measurement Analysis Interpretation	***Conceptual***: context
Empirical study	Westergren [36]	2012	Sweden	Long-term care	Explore staff’s views of participating in action-oriented study circles focused on eating and nutrition	Mixed	PARIHS	Analysis Interpretation	***Relational***: dynamics among evidence, context, facilitation, and implementation success; ***Conceptual***: implementation success
Empirical study	Rycroft-Malone [35]	2013	Multiple European countries	Acute care/hospitals	Determine how the implementation interventions were received and how implementation processes played out	Qualitative	PARIHS	Analysis Interpretation	***Conceptual***: implementation success, ***Conceptual***: individual as a new element; ***Relational***: dynamics among evidence, context, facilitation, and implementation success
Empirical study	Slaughter [44]	2013	Canada	Long-term care	Assess the effect of the sit-to-stand activity on mobility outcomes, the effect of an audit-and-feedback intervention on uptake of the sit-to-stand activity, and the influence of contextual factors	Quantitative	PARIHS	Measurement Analysis Interpretation	***Relational***: relationships between context, facilitation, and implementation success
Empirical study	Watts [48]	2014	USA	US Veterans Affairs	Examine the effectiveness of Veterans Affairs efforts to promote use of evidence-based psychotherapies and evaluate how it impacts implementation	Mixed	PARIHS	Measurement	***Relational***: context and implementation success, facilitation and implementation success
Empirical study	Gagliardi [20]	2014	Canada	Multiple sectors	Explore how context influenced integrated knowledge translation, in three health services programs for cancer screening, diagnosis, and treatment	Qualitative	PARIHS	Interpretation	***Conceptual***: context; ***Conceptual***: implementation success; ***Relational***: context and implementation success
Empirical study	Tierney [39]	2014	UK	Primary care/community care	Evaluate how context affects the process of facilitating evidence-based practice	Qualitative	PARIHS	Interpretation	***Relational***: relationships between facilitation and context
Empirical study	McCullough [42]	2015	USA	US Veterans Affairs	Identify patterns among contextual elements that influence uptake of an anticoagulation initiative	Qualitative	PARIHS	Measurement Analysis Interpretation	***Relational***: sub-elements of context and implementation success
Empirical study	Sandstrom [17]	2015	Sweden	Primary care/community care	Investigate perceptions of decision-makers regarding implementation of evidence-based guidelines	Qualitative	PARIHS	Analysis Interpretation	***Conceptual***: context
Empirical study	Brown [30]	2016	UK	Acute care settings/hospitals	Explore holistic facilitation as an approach to enabling healthcare teams to critically analyze practice and enhance patient care	Qualitative	Both	Interpretation	***Conceptual***: facilitation
Empirical study	Oye [23]	2016	Norway	Long-term care	Examine the influence of leadership when facilitating change	Qualitative	PARIHS	Analysis Interpretation	***Conceptual***: context (leadership)
Empirical study	Mekki [40]	2017	Norway	Long-term care	Explore the interplay between external facilitation and context relative to intervention outcomes	Qualitative	PARIHS	Measurement Analysis Interpretation	***Relational***: relationships between facilitation and context/recipient
Empirical study	Ward [43]	2017	USA	Acute care settings/hospitals	Identify elements related to successful implementation of a quality improvement initiatives	Qualitative	PARIHS	Measurement Analysis Interpretation	***Relational***: relationships between implementation success and evidence/ facilitation/ context
Empirical study	Diffin [38]	2018	UK	Multiple sectors	Investigate components related to the successful implementation of a person-centered intervention in palliative and end-of-life care	Qualitative	PARIHS	Measurement Interpretation	***Relational***: relationships between facilitation and evidence/context
Empirical study	Harvey [34]	2018	UK	Multiple sectors	Examine two different facilitation strategies employed by a national agency to support the implementation of insulin pump therapy	Qualitative	i-PARIHS	Analysis Interpretation	***Relational***: relationships between facilitation and implementation success
Empirical study	Rycroft-Malone [46]	2018	Multiple European countries	Long-term care	Use a realist process to evaluate the process of implementing two facilitation interventions	Qualitative	PARIHS	Measurement Analysis Interpretation	***Relational***: relationships between context, facilitation, and implementation success
Empirical study	Lo [45]	2018	Canada	Long-term care	Explore the effect of clinical educators as facilitators of research use and how it is modified by context	Quantitative	PARIHS	Measurement Interpretation	***Relational***: interactions between context and facilitation in impacting implementation success
Empirical study	Butow [29]	2019	Australia	Acute care settings/hospitals	Document barriers and facilitators to the sustainability of ConquerFear	Qualitative	PARIHS	Analysis Interpretation	***Conceptual***: facilitation
Empirical study	Lachance [27]	2019	Canada	Primary care/community care	Examine mentoring as a type of facilitation supporting evidence use	Qualitative	Both	Intervention Measurement Analysis Interpretation	***Conceptual***: facilitation
Empirical study	Connolly [28]	2020	USA	US Veterans Affairs	Examine how facilitation contributes to successful implementation of healthcare interventions	Qualitative	i-PARIHS	Measurement Analysis Interpretation	***Conceptual***: facilitation
Empirical study	Geerligs [19]	2020	Australia	Acute care settings/hospitals	Explore staff perspectives on the feasibility of strategies supporting the implementation of a clinical pathway	Qualitative	PARIHS	Measurement Analysis Interpretation	***Conceptual***: context; ***Conceptual***: facilitation
Empirical study	Niemeyer Hultstrand [18]	2020	Eswatini (Africa)	Primary care/community care	Evaluate the implementation of the Reproductive Life Plan in disadvantaged communities	Mixed	i-PARIHS	Analysis Interpretation	***Conceptual***: context
Empirical study	Granberg [24]	2021	Sweden	Disability healthcare organizations	Explore managers' experiences of the implementation process when transferring new practices into care	Qualitative	i-PARIHS	Measurement Interpretation	***Conceptual***: context (leadership)
Empirical study	Ritchie [32]	2021	USA	US Veterans Affairs	Explore how an expert transfers facilitation skills to support implementation	Qualitative	i-PARIHS	Analysis Interpretation	***Conceptual***: facilitation
Empirical study	Steffen [33]	2021	USA	Acute care settings/hospitals	Examine pediatric intensive care unit-specific implementation barriers and facilitators	Qualitative	i-PARIHS	Measurement Analysis Interpretation	***Conceptual***: facilitation; ***Conceptual***: recipients
Empirical study	Olmos-Ochoa [31]	2021	USA	US Veterans Affairs	Present a case study that illustrates two distinct elements of facilitation and propose an expanded conceptual framework	Qualitative	i-PARIHS	Measurement Analysis Interpretation	***Conceptual***: facilitation
Empirical study	Gustavson [41]	2021	USA	US Veterans Affairs	Explore fluctuations in barriers to medication treatment for opioid use disorder over the course of a 1-year external facilitation intervention	Qualitative	i-PARIHS	Analysis Interpretation	***Relational***: temporal changes in innovation, recipients, and context in response to facilitation
Empirical study	Lo [47]	2021	Canada	Long-term care	Explore the dynamic relationships between context, facilitation, and research use	Quantitative	PARIHS	Measurement Analysis Interpretation	***Relational***: interactions between context and facilitation in impacting implementation success
Literature review	Watson [14]	2018	na	na	Identify external context constructs impacting implementation of complex evidence-based interventions	Integrative systematic review	Both	Interpretation	***Conceptual***: context
Literature review	Clavijo-Chamorro [16]	2020	na	na	Explore the nurses’ perceptions of context-related facilitators for the application of evidence	Meta-synthesis	PARIHS	Analysis Interpretation	***Conceptual***: context
Literature review	Dryden-Palmer [50]	2020	na	na	Synthesize the literature about the impact of, and the potential relationship between context, complexity, and processes	Realist-informed review	PARIHS	An overall guiding framework	***Relational***: dynamics among context, evidence/innovation, and implementation process
Literature review	Clavijo-Chamorro [25]	2021	na	na	Explore nurse managers' perceptions of the factors supporting the implementation of evidence through leadership	Meta-synthesis	PARIHS	Measurement Analysis Interpretation	***Conceptual***: context (leadership)
Theory/methodology article	Stetler [26]	2011	USA	na	Develop a detailed reference guide to help researchers apply PARIHS	Other (e.g., discussion article)	PARIHS	Measurement Analysis Interpretation	***Conceptual***: evidence; ***Conceptual***: facilitation; ***Conceptual***: implementation success
Theory/methodology article	Kramer [37]	2013	Canada	na	Develop a theory-based knowledge-transfer and exchange method of evaluation (KEME) that synthesizes three theoretical frameworks including PARIHS	Mixed	PARIHS	Measurement Analysis Interpretation	***Conceptual***: implementation success
Theory/methodology article	Pfadenhauer [15]	2017	na	na	Develop a framework for structured/comprehensive conceptualization of context and implementation of complex interventions	Other (e.g., discussion article)	PARIHS	Measurement Analysis Interpretation	***Conceptual***: context; ***Relational***: dynamics among context, intervention, and implementation
Theory/methodology article	Mills [49]	2019	UK	na	Assess logic models used in healthcare research from a complexity perspective	Other (e.g., discussion article)	PARIHS	Measurement Analysis Interpretation	***Conceptual***: context ***Relational***: dynamics among context, intervention, and facilitation

*Notes*: ^a^Application of includes: *Intervention*—(i-)PARIHS was used to guide the development of implementation strategies to deliver a clinical intervention, *Measurement*—(i-)PARIHS was used to inform measurement or data collection, *Analysis*—(i-)PARIHS was used to inform data analysis, *Interpretation*—(i-)PARIHS was used to inform the interpretation or evaluation of study findings

### Conceptual advances of (i-)PARIHS elements and sub-elements

Table 3 summarizes conceptual contributions to (i-)PARIHS elements/sub-elements from 24 articles reviewed.

**Table 3:** Original and advanced conceptualizations of (i-)PARIHS elements and sub-elements

**Evidence/innovation** (shaded cells provide the original conceptualization of *evidence* in the PARIHS framework and *innovation* in the i-PARIHS framework)		
**Conceptualization/sub-elements**		**Sources**
***Evidence*** **in PARIHS**		
• Research • Clinical experience	• Patient experience • Information from local context	Kitson et al. (1998) [1]; Rycroft-Malone (2004) [2]
*Note.* Each sub-element is placed on a low–high continuum		
***Innovation*** **in i-PARIHS**		
• Underlying knowledge sources • Clarity • Relative advantage • Trialability	• Compatibility or contestability (degree of fit with existing practice and values) • Usability	Harvey and Kitson (2016) [11]
*Note.* In i-PARIHS, *innovation* is introduced to represent a broader conceptualization of *evidence*		
**Characteristics of evidence-based practice as a new sub-element of** ***evidence*:**		
• Relative advantage • Observability • Compatibility • Complexity	• Trialability • Design quality and packaging • Costs	Stetler et al. (2013) [26]^a^
*Note.* Particularly relevant in implementation programs targeting a specific evidence-based practice		
**Context** (shaded cells provide the original conceptualizations of *context* in the [i-]PARIHS framework)		
**Conceptualization/sub-elements**		**Sources**
***Context*** **in PARIHS**		
• Receptive context • Culture	• Leadership • Evaluation	Kitson et al. (1998) [1]; Rycroft-Malone (2004) [2]
*Note.* Each sub-element is placed on a low–high continuum		
**C*ontext*** **in i-PARIHS**		
Local level (micro): • Formal and informal leadership support • Culture • Past experience of innovation and change • Mechanisms for embedding change • Evaluation and feedback processes • Learning environment Organizational level (meso): • Organizational priorities • Senior leadership and management support	• Culture • Structure and systems • History of innovation and change • Absorptive capacity • Learning networks External health system level (macro): • Policy drivers and priorities • Incentives and mandates • Regulatory frameworks • Environmental (in)stability • Inter-organizational networks and relationships	Harvey and Kitson (2016) [11]
*Note.* Context is also categorized as *inner context* (local and organizational level) and *outer context* (external health system level)		
**Conceptualization of** ***external/outer context***		
External social networks and embedded communication pathways *Note.* Particularly important for fragmented sectors where lines of information sharing are non-linear and multidirectional		Carlan et al. (2012) [21]^a^
External resources (resource abundance or scarcity):		
• Human resources • Space	• Medicine/equipment/other supplies • Communication/transport	Bergstrom et al. (2012) [22]^a^
*Note.* Particularly relevant in low-income healthcare settings		
Broader organizational capacity or system-level capacity: • Socio-political (clear goals, consensus on goals among stakeholders) • Economic (dedicated funding, resources, and infrastructure) • Social (formal and informal interactions among parties, affiliated or embedded roles, diversity of engaged disciplines) *Note.* Particularly relevant in integrated knowledge translation activities in which researchers, clinicians, and other stakeholders purposefully convene for a particular integrated knowledge translation program		Gagliardi et al. (2014) [20]^a^
Contextual trust • Credibility of external organizations •Staff's sense of certainty about external organizations *Note.* External organizations include entities developing evidence-based guidelines, and entities promoting and funding implementation of evidence-based guidelines.		Sandstrom et al. (2015) [17]^a^ Geerligs et al. (2020) [19]^a^
Societal culture, social systems, or hegemony *Note.* Particularly relevant in implementation of public health interventions		Niemeyer Hultstrand et al. (2020) [18]^b^
**Conceptualization of** ***leadership***		
*Leadership* as a social collective process that has dynamic relationships with team culture, is characterized by diversified styles and diverse actions and operates in multiple ways and on multiple levels in the organization.		Oye et al. (2016) [23]^a^
*Leadership* (of nurse managers) as a facilitator of evidence implementation: • Effective teamwork ○ Communication between managers and staff nurses ○ Foster collaboration • Effective organizational structures ○ Strategic governance (leadership combining management and clinical experience, awareness of the impact of improved outcomes) • Transformational leadership ○ Influence on evidence application (culture of expectations, inspiring a shared vision, sustaining evidence-based practice) ○ Readiness for change among leaders (enabling empowering, focus on teaching and learning, recognition, using resources in management) *Note.* This meta-synthesis expanded conceptualizations of general elements of leadership in PARIHS (teamwork, organization structures, and transformational leadership) by elaborating on features of each element		Clavijo-Chamorro et al. (2021) [25]^a^
“Balance between leadership and management to maximize their influence on the implementation process” as a new element of *leadership* *Note*. This study highlighted the complex interplay between leadership and management and consequences for the implementation process		Granberg et al. (2021) [24]
**Conceptualization of** ***context*** **in new context typologies for particular implementation context**		
External context impacting implementation of **complex interventions:**		Watson et al. (2018) [14]^c^
• Professional influences • Political support • Social climate • Local infrastructure	• Policy and legal climate • Relational climate • Target population • Funding and economic climate	
The Context and Implementation of Complex Interventions (CICI) framework (context impacting implementation of **public health interventions**):		Pfadenhauer et al. (2017) [15]^a^
• Geographical • Epidemiological • Socio-cultural • Socio-economic	• Ethical • Legal • Political	
*Note.* According to the CICI framework, the seven domains of context act on their own and interact with interventions and implementation at macro, meso, and/or micro levels		
Institutional context relevant in **evidence application by nursing professionals:** • Institutional support (leadership) • Multidisciplinary support (teamwork and communication) • Culture of improving quality of care (nursing professionals’ attitudes toward change • Use of research (valuing research, assessment of research results, dissemination of results and experiences).		Clavijo-Chamorro et al. (2020) [16]^a^
**Facilitation** (shaded cells provide the original conceptualizations of *facilitation* in the [i-]PARIHS framework)		
**Conceptualization/sub-elements**		**Sources**
***Facilitation*** **in PARIHS**		Kitson et al. (1998) [1]; Rycroft-Malone (2004) [2]
• Purpose (from task-oriented to holistic-oriented) • Role • Skills and attributes *Note.* Roles and associated skills and attributes are described separately for the two different purposes		
***Facilitation*** **in i-PARIHS**		Harvey and Kitson (2016) [11]
Facilitation is conceptualized as the active ingredient that activates implementation through assessing and responding to characteristics of the innovation and the recipients within their contextual setting. Facilitation role and process are specified for novice, experienced and expert facilitators.		
**Conceptualization of** ***facilitation*** **with a focus on facilitation processes/strategies**		
*Facilitation* as a deliberate implementation intervention: • Comprises deliberate methods or techniques facilitators use to facilitate evidence-based practice uptake • Is developed based on pre-implementation diagnostic assessments of the organizational context and staff's perceptions of the intervention • Promotes intervention/service fit and intervention/patient fit which is fundamental for improving sustainability. *Note.* Particularly applicable when implementing a specific targeted evidence-based practice or clinical intervention		Stetler et al. (2010) [26]^a^ ; Geerligs et al. (2020) [19]^a^; Butow et al. (2019) [29]^a^
*Facilitation* as specific facilitative actions and strategies:		Steffen et al. (2021) [33]^b^
• Plan strategies • Educate strategies	• Quality management strategies • Other strategies	
*Note.* The authors detail specific facilitative actions under each category of strategies		
Holistic-oriented facilitation: • Creating a psychologically safe space • Establishing capacity-building mentoring relationships *Note.* Particularly important for ‘weak context’ environments		Brown et al. (2016) [30]^c^; Lachance et al. (2019) [27]^c^
**Conceptualization of facilitation with a focus on facilitation roles**		
Internal & external *facilitators*		Connolly et al. (2020) [28]^b^
• Internal facilitators offer localized knowledge about needs, policy, and culture. Skills include project management, team and process skills, and influencing and negotiating skills. Personal characteristics include leadership and emotional intelligence.	•External facilitators serve as an expert, consultant, model, and educator, providing concrete advice and direction on intervention content and implementation processes.	
*Note.* Dynamics between external and internal facilitation is an important aspect of the conceptualization, which significantly vary across projects and settings, and evolve as facilitation progresses.		
Active versus passive *facilitation* (specifically pertinent to external facilitation roles of national agencies) • Active facilitation applied by national agencies involves designated external facilitators who use project management methods to structure a site-specific implementation process for individual sites. • Passive facilitation involves raising public awareness of the existence of an innovation through web-based facilitation methods. This method could cause uncertainty and disagreement among stakeholders around the benefits of the innovation and its implementation.		Harvey et al. (2018) [34]^b^
Transferring facilitation skills from expert to novice facilitators: • Direct skill transfer ○ Active methods (teaching, modeling, coaching) ○ Participatory methods (working together, providing consultation) • Learning supports ○ Cognitive learning supports ○ Psychosocial learning supports ○ Self-learning promotion ○ Structural supports		Ritchie et al. (2021) [32]^b^
*Facilitation intensity* and *facilitator resilience* as new sub-elements of facilitation: • Facilitation intensity: the quantitative and qualitative measure of the volume of tasks and activities of facilitation • Facilitator resilience: facilitator’s ability to cope with and adapt to the complexities of facilitation *Note.* Facilitation intensity and facilitator resilience are conceptualized based on perceptions and experiences of facilitators themselves and reflect psychological impacts of facilitation processes on facilitator effectiveness and implementation success		Olmos-Ochoa et al. (2021) [31]^b^
**Implementation success** (shaded cells provide the original conceptualization of *implementation success* in the i-PARIHS framework)		
**Conceptualization/sub-elements**		**Sources**
***Implementation success*** **in the i-PARIHS framework** • Achievement of agreed implementation/project goals • The uptake and embedding of the innovation in the practice context • Individuals, teams, and stakeholders are engaged, motivated, and own the innovation • Variation related to context is minimized across implementation settings		Harvey and Kitson (2016) [11]
**Conceptualization of** ***implementation success*** **in particular implementation contexts**		
*Implementation success* in the context of implementing a specific targeted evidence-based practice or clinical intervention: • Realization of the implementation plan • Evidence-based practice innovation uptake (desired changes in practice that are congruent with the evidence-based practice) • Achievement and sustainment of patient and organizational outcomes *Note.* Iterative theory-driven formative/process evaluations are recommended to measure implementation success, especially “realization of implementation plan”		Stetler et al. (2010) [26]^a^ ; Rycroft-Malone et al*.* (2013) [35]^a^
*Implementation success* in the context of implementing holistic facilitation		Westergren (2012) [36]^a^
• Instrumental outcomes ○ Improved system performance (care outcomes) ○ Measurable changes in patient outcomes (health outcomes)	• Conceptual outcomes ○ Professional development ○ Enhanced organizational context	
**Conceptualization of broad** ***knowledge translation outcomes***		
*Knowledge translation outcomes* in integrated knowledge translation: • Health service outcomes (accomplish professional goals, conceptual use of research, instrumental use of research) • Social outcomes (researcher-research user interactions, diversity of disciplines, mutual learning/understanding) • Research outcomes (efficiency, quality, relevance, accelerated progress, inform new research, publications/reports)		Gagliardi et al. (2014) [20]^a^
*Knowledge use outcomes as knowledge translation outcomes* • Conceptual use of knowledge (research is used to gradually frame the understanding of an issue; also called enlightenment or indirect use of knowledge) • Instrumental use of knowledge (research is used to design a new policy, program, or procedure; also called structural, problem-solving, or direct use of knowledge) • Strategic use of knowledge (research is used to justify a course of action already decided upon; also called political, tactical, or symbolic use of knowledge)		Kramer et al*.* (2013) [37]^a^

**Recipients** (shaded cells provide original conceptualizations of *implementation success* in the i-PARIHS framework)		
**Conceptualization/sub-elements**		**Sources**
***Recipients*** **in i-PARIHS**		Harvey and Kitson (2016) [11]
Recipients (people who are affected by and influence implementation at both the individual and collective team level) characteristics:		
• Motivation • Values and beliefs • Goals • Skills and knowledge • Time, resources, support	• Local opinion leaders • Collaboration and teamwork • Existing networks • Power and authority • Presence of boundaries	
Individual factors as an explicit additional element of PARIHS		Rycroft-Malone et al. (2013) [35]^a^
• Capability • Capacity • Motivation • Resilience • Acceptability • Feelings	• Knowledge and beliefs about the innovation • Position and fit within the organization/social system • Approach to decision-making	
*Professional expectations and requirements* as new sub-elements of recipients *Note.* Relevant because professionals are generally expected, not required, to implement evidence-based practice		Steffen et al. (2021) [33]^b^

Notes

^a^The article focused on the PARIHS framework

^b^The article focused on the i-PARIHS framework

^c^The article focused on both the PARIHS and i-PARIHS frameworks

### Twelve articles advanced the conceptualization of context

Three articles proposed new context typologies with particular applicability in certain implementation situations, including implementation of complex interventions [14], implementation of public health interventions [15], and evidence application by nursing professionals [16].

Six empirical articles proposed conceptualizations of outer/external context [17–22]. Emphases on sub-elements of outer/external context depended on implementation contexts, such as external resources (abundance or scarcity) in low-income healthcare settings [22]; capacity of researchers, clinicians, and other stakeholders for integrated knowledge translation programs [20]; societal culture for implementing public health interventions [18]; credibility of external entities responsible for developing or promoting clinical guidelines to implement a specific clinical guideline [17, 19]; and social networks and communication pathways with external entities in sectors such as the construction industry, where lines of information-sharing were fragmented and multidirectional [21].

Three empirical articles [23–25] expanded the conceptualization of *leadership* (one sub-element of *context*) beyond the low–high continuum in the PARIHS framework. One article suggested conceptualizing *leadership* as a versatile, dynamic construct that operates in multiple ways and on multiple levels in an organization and evolves over implementation intervals [23]. One article detailed sub-elements under each general element of *leadership* (teamwork organizational structure, transformational leadership) in the PARIHS framework [25]. One article suggested “balance between leadership and management to maximize their influence on the implementation process” as a new element of leadership [24].

### Ten articles advanced the conceptualization of facilitation

Articles extended the conceptualization of *facilitation* as roles, processes, or strategies that facilitate the achievement of implementation goals [19, 26–34]. Among articles focusing on facilitation processes or strategies, three suggested facilitation as a deliberate implementation intervention targeting evidence–context fit [19, 26, 29]. Two articles discussed holistic-oriented facilitation through creating a psychologically safe space [30] or establishing capacity-building mentoring relationships [27]. One article described facilitation as four categories of strategies—planning, restructuring, education, and quality management—with specific facilitative actions under each category [33].

Among articles focusing on facilitation roles, one elaborated the conceptualization of facilitation by distinguishing between external and internal facilitation roles and the dynamics between them [28]. One article compared active and passive facilitation roles of national agencies to promote innovation uptake [34]. One article described the role transfer process from expert to novice facilitators [32]. One article suggested two new *facilitation* sub-elements, facilitation intensity and facilitator resilience, conceptualized based on the perceptions and experiences of facilitators themselves [31].

### Five articles explicitly discussed the conceptualization of implementation success

Three articles focused on outcomes in specific implementation situations such as implementing a particular targeted evidence-based practice (EBP) [26, 35] and implementing holistic-oriented facilitation [36]. Successful implementation of an EBP is suggested to be reflected in three aspects: the implementation plan and its realization, EBP innovation uptake, and the achievement of patient and/or organizational outcomes [26, 35]. When implementing holistic-oriented facilitation, implementation outcomes encompass both instrumental outcomes (improvement in system performance and patient outcomes), and conceptual outcomes (professional development and improvement in organizational context).

Two articles addressed broader knowledge translation outcomes [20, 37]. For example, outcomes in an integrated knowledge translation project were suggested to include health service outcomes (e.g., accomplished professional goals and use of research), social outcomes (e.g., collaborations between researchers and research users), and research outcomes (e.g., enhanced quality/relevance of research) [20]. One article expanded the conceptualization of knowledge use, categorizing it into conceptual, instrumental, and strategic use of knowledge [37].

Finally, one article expanded *the conceptualization of evidence* and suggested EBP characteristics as a new sub-element of evidence when applying the PARIHS framework in implementing programs that target a specific EBP [26]. Two studies discussed *the conceptualization of recipients* [33, 35], suggesting individual factors as a new element [35] or “professional expectations and requirements” as a new sub-element of *recipients* [33].

### Advancing understanding of relationships among (i-)PARIHS elements

Table 4 describes contributions to relational aspects of the (i-)PARIHS framework. Four articles provided insights into dyadic relationships (relationships between two variables) of *facilitation* with other (i-)PARIHS elements. Three of those articles discussed the dyadic relationship of *facilitation* with *evidence* [38], with *context* [38–40], or with *recipients* [40]. One article discussed temporal changes in other elements in response to *facilitation* [41].

**Table 4:** Relational contributions

Relational focus		Key findings from individual articles	Sources
**Relationships between** ***facilitation*** **and other (i-)PARIHS elements**			
**Dyadic relationship between** ***evidence*** **and** ***facilitation***		The linkage between *facilitation* and *evidence* occurs as internal facilitators communicate the evidence to colleagues and this connection is described as ‘legitimizing the evidence’ and ‘informed advocacy.’	Diffin et al. (2018) [38]
**Dyadic relationship between** ***context*** **and** ***facilitation***		Existing contextual factors help and hinder the facilitation process directly and indirectly. The strength of the influences of contextual factors on facilitation process fluctuates over the implementation intervals.	Diffin et al. (2018) [38]; Mekki et al. (2017) [40]
		There is a complex interplay between *facilitation* and *context*, where macro and micro contextual factors contribute to tensions associated with facilitation. Tensions can have both positive and negative components.	Tierney et al. (2014) [39]
**Dyadic relationship between** ***recipients*** **and** ***facilitation***		Recipients impact facilitation at two stages. Recipients’ learning skills (creative and analytical reasoning) and motivation influence the early stage of facilitation which concerns acquisition of new knowledge. Recipients’ application of new knowledge impacts the next stage of facilitation which is intended to put the agreed decisions into action.	Mekki et al. (2017) [40]
**Temporal changes in i-PARIHS elements in response to** ***facilitation***		*Facilitation* changes barriers related to *innovation, recipients,* and c*ontext* over time as the facilitation processes progress. *Innovation, recipients,* and c*ontext* respond to *facilitation* at different time points and to different degrees.	Gustavson et al. (2021) [41]
**Relationships between (i-)PARIHS elements (evidence, context, facilitation) and implementation outcomes**			
**Relative importance of (i-)PARIHS elements/sub-elements** **regarding necessity and sufficiency in impacting implementation outcomes**	***Evidence*** **is necessary but not sufficient to influence evidence-based practice uptake**	When there is a strong belief in evidence, strength in at least one contextual element (leadership, culture, or evaluation) is needed for uptake to occur; when there is limited belief in the evidence, strengths in the contextual elements unexpectedly reinforced the resistance to change.	McCullough et al. (2015) [42]
		When the evidence is robust, uptake of the evidence is mainly determined by individual factors (practitioners’ and patients’ behaviors, attitudes, emotional responses), inter-professional functioning, and the organization’s existing systems and processes.	Rycroft-Malone et al. (2013) [35]
	***Leadership*** **is necessary and sufficient to positively implementation outcomes**	Leadership exerts the greatest influence on implementation processes in which favorable culture and evaluation influence facilitation only when the clinical leaders take an active role.	Mekki et al. (2017) [40]
		Leadership and capacity (infrastructure, funding, clear goals), even in the absence of a strong knowledge translation culture, are sufficient to achieve tangible implementation outcomes.	Gagliardi et al. (2014) [20]
	**The impacts of** ***facilitation*** **and** ***context*** **on implementation outcomes depend on the phases of implementation**	Phases of implementation include problem identification, intervention design, development of implementation strategy, and reinforcement. *Leadership* (not other context sub-elements) influences the phase problem identification. *Facilitation* sub-elements (role of facilitator, goal/purpose, character/style, and skills/attributes) influence all phases of implementation.	Ward et al. (2017) [43]
**(i-)PARIHS elements/sub-elements interact in affecting implementation outcomes**	***Facilitation*** **contributing to implementation outcomes is contingent on** ***context***	Facilitation with broad training goals in clinics with highly organized systems (such as mental health care) is negatively associated with evidence-based practice implementation.	Watts et al. [48]
		Facilitation practices can yield positive impacts on research use in settings with low ratings for leadership and culture but where a proficient evaluation/feedback process is in place.	Lo et al. (2018) [45]
		Facilitation practices influencing research use depends on different combined patterns of context sub-elements (e.g., leadership, culture, evaluation, structural resources, organizational slack)	Lo et al. (2021) [47]
	**Interactions among** ***context*** **sub-elements can produce synergy or compensation**	The combination of strong context sub-elements (e.g., leadership, teamwork, and communication) magnify the effect of single elements, and a strong context sub-element may compensate for other weaker ones.	McCullough et al. (2015) [42]
	***Facilitation*** **and** ***context*** **are complementary**	Facilitation increases innovation uptake in settings with an initially unreceptive context. In settings with an initially strong, receptive context, innovation uptake is apparent despite the absence of facilitation interventions.	Slaughter and Estabrooks (2013) [44]
**What types of** ***facilitation*** **work (or do not work), for whom, how, why, and in what circumstances, in improving implementation outcomes**	***Facilitation*** **mechanisms grounded in organizational learning frameworks**	Holistic facilitation based on action-oriented collaborative learning approaches fosters the development of a learning organization in which socio-culturally mediated knowledge spread occurred, which ultimately facilitated evidence implementation. Specifically, holistic facilitation promotes the re-creation of knowledge (where evidence is reshaped) as professionals exchange their perspectives and work collaboratively toward setting up goals; holistic facilitation prioritizes professionals’ needs and motivation, and contextual components that are related to evidence implementation.	Westergren (2012) [36]
	**Context–mechanism–outcome configurations grounded in realist approaches**	Three interrelated mechanisms in which facilitation impacts implementation success has been identified: aligning the project with the needs and expectations of facilitators and the organization, prioritizing the project in the organization, and fostering collective engagement by staff and stakeholders. The three mechanisms together reinforce organizational learning and facilitators’ enactment of their roles over time, which led to positive outcomes. Whether a mechanism is triggered is contingent on context (e.g., outside regulatory environment, stability of the internal context, and relationships between internal facilitators and managers).	Rycroft-Malone et al. (2018) [46]
**Theorizing the dynamism among PARIHS elements drawing on complex systems perspectives and broader theoretical models in implementation science**			
Complexity is an inherent characteristic of each PARIHS element and also emerges as a component of the dynamic (non-linear, non-static, and adaptive) relationships among these elements		The following general dynamism among complex interventions, context, facilitation/facilitative implementation process, and implementation outcomes has been identified: • Complexity is an inherent characteristic of all the elements. • Complexity also emerges as a component of the dynamic relationships among these elements. • A complex intervention is formed by its interaction with the context at the macro, meso, and micro levels. • Facilitation strategies respond to a full spectrum of contextual factors (from the inner to outer context), which will in turn reshape the intervention. • Inner and outer context is never static, and the implementation of facilitation strategies and assimilation of the intervention generally alter the context. • Changes in contextual factors should be evaluated as an outcome, as referred to as proximal contextual outcomes, besides distal intervention outcomes. • Implementation process plays as a stand-alone component (described as socially mediated processes that are frequently qualified as facilitative). • Elements respond to one another intentionally or organically, and their relationships evolve as the intervention is moved through the phases of preparation, introduction, activation, and integration toward adoption.	Pfadenhauer et al. (2017) [15]; Mills et al. (2019) [49]; Dryden-Palmer et al. (2020) [50]

Eleven articles on individual empirical studies extended beyond dyadic considerations to more complex relationships linking core (i-)PARIHS elements (*evidence*, *context*, *facilitation*, *recipients*, and their sub-elements) to implementation outcomes [20, 35, 36, 40, 42–48]. These articles addressed three broad relational questions:

(i) What is the relative importance of the core (i-)PARIHS elements/sub-elements in affecting implementation outcomes? The articles identified that *evidence* is necessary but not sufficient [35, 42], *leadership* is necessary and sufficient [20, 40], and impacts of *facilitation* and *context* on implementation outcomes depend on the phase of implementation [43];
(ii) How do (i-)PARIHS elements/sub-elements interact with one another in affecting implementation outcomes? Articles identified that *facilitation’s* contribution to implementation outcomes or research use is contingent on *context* [45, 47, 48], that interactions among *context* sub-elements can produce synergy or compensation [42], and that *facilitation* and *context* are complementary [44].
(iii) What types of facilitation do or do not work, for whom, how, why, and in what circumstances, in improving implementation outcomes? Articles identified that *facilitation* mechanisms can be usefully grounded in organizational learning frameworks [36] and that context-mechanism-outcome configurations are feasibly grounded in realist approaches [46].

Using research synthesis approaches, three articles integrated (i-)PARIHS with complex system perspectives and broader theoretical models in implementation science to describe the dynamism among (i-)PARIHS elements, particularly in the context of implementing complex interventions [15, 49, 50]. These reports identify complexity is an inherent characteristic of each (i-)PARIHS element and also as a component of the dynamic relationships among these elements. The dynamism is reflected by the non-linear, non-static, and adaptive relationships among the core elements.

## Discussion

We undertook this review to determine how the conceptualizations of (i-)PARIHS elements/sub-elements, the relationships between them, and dynamics among them have advanced since publication of the critical review by Helfrich and colleagues [9]. We observed an increasing number of studies that applied (i-)PARIHS explicitly to guide implementation research at different stages, from developing implementation interventions to evaluating implementation outcomes. Although much of the (i-)PARIHS literature confirmed what is set out in the (i-)PARIHS framework, we identified some articles that offered conceptual and relational advances. Here we discuss contributions added by those articles to the (i-)PARIHS framework. We also offer directions to further advance the frameworks.

### Steps toward conceptual clarity and comprehensiveness

The articles included in this review addressed conceptual expansion of all the core (i-)PARIHS elements with most emphasis placed on *context* (particularly *outer context* and *leadership*)*, facilitation,* and *implementation successes.* Some conceptual limitations noted for the original PARIHS framework were resolved with the revised i-PARIHS framework [11, 12], but we highlight persisting concerns about conceptualization of some concepts that need to be further developed.

Although the concepts of *evidence/innovations* and *recipients* were not discussed frequently, we see the introduction of ‘implementable’ characteristics of *evidence* in the article by Stetler et al. [26] as promising, along with Rycroft-Malone et al.’s [35] suggestion of including individual recipients’ characteristics as a new element. Findings from these two articles offered implications for developing the revised i-PARIHS framework where *evidence* is conceptualized as a new concept *innovation* and *recipients* is added as a stand-alone element [26, 35]. Explicit definitions of and differentiation between various implementable characteristics of *evidence/innovations* have rendered this concept more measurable. Inclusion of *recipients* as a core element has improved the explanatory power of the framework. Nevertheless, we note that these two concepts received less attention than other (i-)PARIHS concepts in diagnostic assessments or process evaluations in implementation studies, and these concepts’ operational definitions remain to be developed.

The most obvious advance in conceptual development relates to the concept of *context*. A number of articles usefully distinguish between the influences of *inner* and *outer context* and consider the importance of outer context to implementation [14, 15, 17–22]. This work is commendable and necessary. However, the considerable variation in how *outer context* was conceptualized (ranging from the influences of specific extra-organizational entities such as guideline-producing organizations to various levels of ‘contextual trust’ ascribed to broad political and economic characteristics), and the under-developed evaluative approaches of *outer context*, make it difficult at this stage to determine its effects on implementation.

The conceptualization of *leadership* (one sub-element of *context*) is discussed widely. Several articles suggest conceptualizing *leadership* as a multifaced construct beyond a low–high continuum [14–16, 23]. Despite the conceptual insight, we note a lack of empirical studies that explored typologies of leadership to address the issues associated with the low–high-continuum approach. Another insight into conceptualization of *leadership* is the suggestion of describing leadership as perceived by clinical leaders themselves (such as care managers). This could uncover an often-overlooked aspect of leadership—the balance between management and leadership and the dual roles of a clinical leader in management tasks and in leading changes [24, 25]. This has not been studied widely. We suggest future research to further examine leadership from the lens of clinical leaders and differentiate between leadership and management. Moreover, other sub-elements of *context* at the local (micro) or organizational (meso) level, such as culture, learning environment, and evaluation and feedback process, need to be further studied. These sub-elements of *context* are central to implantation research and yet to be explicitly conceptualized and operationalized.

Expansion of the concepts of *facilitation* and *implementation success* are major contributions to the literature over the past decade. We found it helpful to distinguish between different types of implementation contexts when defining *facilitation* and *implementation success*, as was attempted in several articles we reviewed. These articles examined two types of implementation contexts. One implemented a targeted EPB or clinical intervention through explicit task-oriented facilitation strategies that aimed for specific clinical outcomes [19, 26, 29, 35]. The other focused on holistic-oriented facilitation that aimed to generally enhance organizational context and staff’s attitudes and behaviors [27, 30, 36]. *Facilitation* and *implementation success* were conceptualized differently: narrow and project-specific in the first context versus broad and organization-wide in the second.

The conceptualization of *facilitation* has expanded beyond internal facilitation roles and processes to address the dynamics between internal and external facilitation [28] and the process of transferring facilitation skills from expert to novice facilitators [32]. This research area needs more attention, given the growing application of complex innovations that involve multiple players from internal and external organizations and facilitators with different levels of facilitation skills. Another important research agenda is to understand the concept of facilitation from the perspectives, perceptions, and experiences of facilitators themselves, which will provide a different lens to examine facilitation and its relationship to implementation outcomes [31].

Similarly, the concept of *implementation success* has expanded to include conceptualizations at different levels of analysis: broadly at system or organization levels and more narrowly at the level of work units or projects [20, 37]. The literature advocates for comprehensive measures of outcomes at different stages of implementation rather than merely outcome measures at the final stage. Theory-driven formative or process evaluations were frequently recommended [20, 26, 35–37]. We suggest that classifying *implementation success* to incorporate levels of analysis and implementation stages would facilitate conceptualization of the (broad) concept of *implementation success*.

## Relational insights

Several articles explicitly attempted to improve our understanding of the complex relationships among (i-)PARIHS elements/sub-elements. Nonetheless, more empirical research is needed to theorize the relationships through actively using empirical data and carefully clarifying the range of specific instances to which findings apply. As suggested by Kislov et al. in 2019 [51], knowledge will advance with *theoretical informative research* (in addition to mere *theoretical informed research*) based on theorizing practices, instead of using the (i-)PARIHS framework as heuristic tools. Here we discuss theoretical and methodological considerations for researchers to further theorize relationships between (i-)PARIHS elements/sub-elements.

Articles articulating dyadic relationships of *facilitation* with either of the other three elements (*evidence*/*innovation*, *context*, *recipients*) shed some light on the role of *facilitation* as the active ingredient in integrating other elements through bidirectional (rather than unidirectional) relationships unfolding at different levels (macro, meso, and micro) and at different stages (temporal dynamic) [38–40]. However, research was lacking that thoroughly examined relationships of *facilitation* with all other elements. In addition, a lack of longitudinal quantitative designs hindered a systematic understanding of the temporal dynamics among the (i-)PARIHS elements and how they correspond to different stages of an implementation process. More investigation at the sub-element level is also needed to understand more granular relationships, such as how links of *facilitation* to *leadership* (one sub-element of *context*) might differ from links of *facilitation* to *evaluation* (another sub-element of *context*).

Some articles [20, 35, 36, 40, 42–48] extended beyond dyadic relationships to more complex relationships that linked implementation outcomes with (i-)PARIHS core elements (*evidence*, *context*, *facilitation*, *recipients*, and their sub-elements). These articles provided three important implications for future research to establish the relational propositions. *First*, it continues to be important for future studies to systematically examine the necessity and sufficiency of (i-)PARIHS elements/sub-elements in relation to implementation outcomes. More empirical research is needed to verify the findings synthesized from these articles on the necessity and sufficiency of some elements or sub-elements. For example, *evidence* (specifically belief in quality of evidence) was viewed as a necessary but not sufficient factor [35, 42], and *leadership* (one sub-element of *context*) was found as a necessary and sufficient determinant of positive implementation outcomes [20, 40]. The primacy of other elements/sub-elements also needs investigation. Studies we reviewed that examined necessary and sufficient factors for implementation success all applied qualitative methods, but we recommend future quantitative research to systematically examine primary ‘difference makers’ in an implementation process. Advances in quantitative methods such as configurational comparative analysis have enabled researchers to quantify the causal complexity in implementation research [52].

*Second*, an important research agenda is to clarify the interactive relationships among (i-)PARIHS elements/sub-elements and the effects of these interactions on implementation outcomes. Interactions among elements and sub-elements may exert complementary, synergistic, or buffering effects on implementation outcomes [42, 44, 45, 47, 48]. Some quantitative studies we reviewed offer good examples of using basic and advanced statistical interaction analyses to quantify the interactive complexities among (i-)PARIHS elements/sub-elements [45, 47]. However, much more empirical research is still needed to uncover underlying casual structures that explain why the interactions occur. More advanced quantitative methods such as structural equation modeling techniques might be used to pursue advanced relational studies [53].

*Third*, research focusing on mechanism-based explanations has enormous scientific and practical value, given the increasing complexity and variability of facilitation/implementation interventions that unfold in diverse and changing contexts. Two articles [36, 46] illustrate that facilitation influences implementation outcomes through more than one mechanism. Complexities embedded in facilitation mechanisms call for integration of (i-)PARIHS with other theoretical and methodological perspectives and tools to provide mechanism-based explanations. For example, organizational learning frameworks [36] and a realist-informed approach [46] were used in combination with the (i-)PARIHS framework to explain what/how/why/for whom a facilitation intervention works and to develop context–mechanism–outcome configurations (referred to as realist-informed contingent explanations). Only a few studies [36, 46] offer mechanism-based explanations. This form of research requires in-depth, extensive process evaluations and often includes modeling techniques such as structural equation modeling and configurational comparative analysis to draw quantitative conclusions. Nonetheless, process evaluations and mechanism-based explanations offer great promise. Outcome measures alone may lead to false negative conclusions on the effectiveness of facilitation/implementation interventions and effects of clinical interventions or evidence-based practice being implemented.

Some articles integrated the (i-)PARIHS framework with complex system perspectives and broader theoretical knowledge in implementation science and other disciplines [15, 49, 50]. These articles strengthened our understanding of the dynamism among (i-)PARIHS elements that unfold at multiple levels of context (macro, meso, micro) and at different stages of an implementation process [15, 49, 50]. These articles developed more comprehensive models that varied in levels of abstraction and generalizability, from a logic model/program theory [49] to a conceptual model that synthesized and consolidated existing theoretical and empirical findings [15, 50]. These integrated models might support developing mid-range theories, which are seen as critical for implementation science because they generate testable propositions and provide practically adequate explanations applicable to a specified set of contexts [51]. However, the utility of the integrated models generated in these articles needs validation with empirical data.

## Limitations of this review

Some limitations of this critical review must be noted. First, we included only peer-reviewed published research in English. We might have omitted important work applying the (i-)PARIHS framework that was published as gray literature or in different languages. Second, our review focused only on articles and studies applying the (i-)PARIHS framework. Implications of the conceptualizations of and relationships among the core implementation constructs are not generalizable broadly for implementation science. Researchers who seek to elaborate the conceptualization of these the core implementation constructs more generally may also want to draw on other conceptual and theoretical models in implementation science or other disciplines, as suggested by Nielsen [54]. Third, the scope of included articles was wide and the applicability of broad findings to specific healthcare settings is difficult to determine. Finally, although a critical interpretive synthesis approach (which was initially developed to synthesize evidence related to concept and theory development from both qualitative and quantitative research) was better suited for the purpose of this review than other synthesis methods, this method is not without limitations. One limitation of a critical interpretive synthesis that has been noted is the low reproducibility of its protocols and findings as a different team using the same set of articles might produce different synthesis results and different interpretation of findings [13]. We used a team consensus-based decision-making approach and worked to maintain transparency in documenting study steps to mitigate the limitation of low reproducibility.

## Conclusions

We reviewed the last decade of implementation research articles predicated on the (i-)PARIHS framework. We used critical interpretive synthesis to identify conceptual and relational advances in the frameworks. We found conceptual expansion of all core (i-)PARIHS elements with most emphasis on *context* (particularly *outer context* and one of the key elements of context—*leadership*)*, facilitation,* and *implementation success.* Articles also gave insights into the complex relationships and relational dynamism among these elements, characterized as contingent, interactive, multilevel, and temporal effects. We propose several directions to further advance the (i-)PARIHS framework:

1. We suggest that conceptualization of core (i-)PARIHS elements (particularly *evidence/innovation* and *recipients*) need to be further developed by specifying conceptual and operational definitions of underlying sub-elements.
2. Relationships among (i-)PARIHS elements/sub-elements need to be further elaborated through empirical studies that consider the situational contingencies for relationships.
3. To provide theoretically and practically meaningful indications, research needs to address relational and causal complexities. This will require examining necessity and sufficiency of (i-)PARIHS elements/sub-elements in relation to implementation outcomes, interactions among elements, and mechanism-based explanations.
4. More quantitative methods, such as configurational comparative analysis [52] and structural equation modeling techniques [53], may be used to quantify causal complexities.

## Supplementary Information

**Additional file 1: Supplementary file 1.** Search strategies in each database

## Data Availability

Not applicable.

## Abbreviations

PARIHS: The Promoting Action on Research Implementation in Health Services (PARIHS) framework
PARIHS: The Integrated Promoting Action on Research Implementation in Health Services (PARIHS) framework
(i-)PARIHS: The Promoting Action on Research Implementation in Health Services (PARIHS) framework or the Integrated Promoting Action on Research Implementation in Health Services (PARIHS) framework
EPB: Evidence-based practice
