# Supplementary figures and images for: Conceptual and relational advances of the PARIHS and i-PARIHS frameworks over the last decade: a critical interpretive synthesis
Source: Implement Sci. 2022 Dec 7;17:78. doi: 10.1186/s13012-022-01254-z (PMC9730581; doi:10.1186/s13012-022-01254-z)

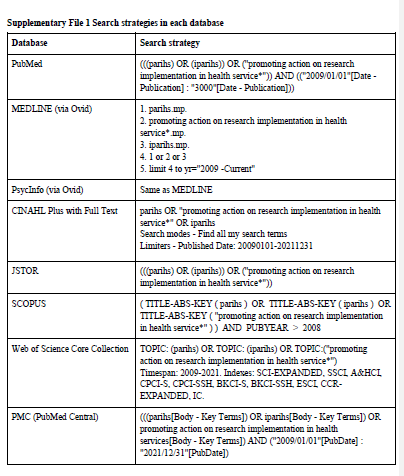

Supplement: Supplementary file 1 — Additional file 1: Supplementary file 1. Search strategies in each database [file 13012_2022_1254_MOESM1_ESM.docx]
